# Impact of Oats on Appetite Hormones and Body Weight Management: A Review

**DOI:** 10.1007/s13668-023-00454-3

**Published:** 2023-02-15

**Authors:** Amna Shehzad, Roshina Rabail, Seemal Munir, Hamza Jan, Diego Fernández-Lázaro, Rana Muhammad Aadil

**Affiliations:** 1 National Institute of Food Science and Technology, University of Agriculture, Faisalabad, 38000 Pakistan; 2 Department of Clinical Nutrition, Nur International University, Lahore, 54950 Pakistan; 3 Departamento de Biología Celular, Genética, Histología y Farmacología, Facultad de Ciencias de la Salud, Campus de Soria, Universidad de Valladolid, Soria, 42004 Spain; 4 Grupo de Investigación Reconocido “Neurobiología”, Facultad de Medicina, Universidad de Valladolid, Valladolid, 47005 Spain

**Keywords:** Obesity, Oat, Beta-glucan, Energy regulation, Satiety hormones, Weight management

## Abstract

**Purpose of Review:**

This study aims to review the hunger hormones in obesity management and the impact of oats in regulating these hormones for hunger suppression and body weight management. In this review, the impact of various edible forms of oats like whole, naked, sprouted, or supplemented has been investigated for their appetite hormones regulation and weight management.

**Recent Findings:**

The onset of obesity has been greatly associated with the appetite-regulating hormones that control, regulate, and suppress hunger, satiety, or energy expenditure. Many observational and clinical studies prove that oats have a positive effect on anthropometric measures like BMI, waist circumference, waist-to-hip ratio, lipid profile, total cholesterol, weight, appetite, and blood pressure. Many studies support the concept that oats are rich in protein, fiber, healthy fats, Fe, Zn, Mg, Mn, free phenolics, ß-glucan, ferulic acid, avenanthramides, and many more. Beta-glucan is the most important bioactive component that lowers cholesterol levels and supports the defense system of the body to prevent infections. Hence, several clinical studies supported oats utilization against obesity, appetite hormones, and energy regulation but still, some studies have shown no or little significance on appetite.

**Summary:**

Results of various studies revealed the therapeutic potentials of oats for body weight management, appetite control, strengthening the immune system, lowering serum cholesterol, and gut microbiota promotion by increased production of short-chain fatty acids.

## Introduction

World Health Organization (WHO) described overweight and obesity as a condition characterized by excessive or abnormal fat accumulation that enhances health risks [1]. It is considered a major public health problem and is the fifth worldwide leading cause of death. The WHO predicted that lifestyle diseases will cause 30% of deaths by 2030, which can be prevented by addressing and identifying risk factors that are related to them and by applying behavioral involvement policies [2]. The global prevalence of overweight and obesity has increased since 1980, with one-third of the global population being categorized as overweight or obese by a rough estimate. Regardless of socioeconomic level, geographic location, or ethnicity, obesity rates have increased in all age groups and genders; however, older people and women are more prone to obesity. Although the absolute prevalence rates of overweight and obesity varied widely among regions and countries, this pattern remained consistent. The body mass index (BMI) is often used in epidemiological research to identify overweight and obesity, although it has limited sensitivity due to considerable inter-individual variability in the percentage of body fat, which is partially based on age, gender, and ethnicity. Asians with the same BMI have a larger percentage of body fat than Caucasians [3]. BMI is computed using height and weight using the Quetelet equation (body weight in kg/height in m^2^) and is classified into five groups according to WHO criteria: Normal weight (18.5–24.99 kg/m^2^), overweight (25.0–29.99 kg/m^2^), obesity grade I (30.0–34.99 kg/m^2^), obesity grade II (35–39.99 kg/m^2^), and obesity grade III (> 40 kg/m^2^) In COVID-19 patients [4].

According to epidemiological research, 35% of people in 2008 were overweight, as the worldwide obesity prevalence increased dramatically between 1980 and 2008. In 2008, 10% of males and 14% of females over the world were obese [5]. The growth in BMI was accelerated throughout east and south Asia for both sexes, but particularly for boys in Southeast Asia. Obesity rates in females climbed from 0.7% in 1975 to 5.6% in 2016, while obesity rates in boys increased from 0.9% in 1975 to 7.8% in 2016. In the same year, there were 50 million obese females and 74 million obese males in the world [6]. This alarming scenario of increasing obesity and overweight prevalence has directed human efforts to find out easily applicable dietary and lifestyle salvation tools. As a result, this review article has made an attempt to focus on the function of hunger hormones in obesity management and the weight-lowering potential of oats ingested as cereal in obesity. For this purpose, the latest available scientific literature from 2018 to date with keywords: “appetite hormones,” “Leptin,” “ghrelin,” “obesity,” “overweight,” “cholecystokinin,” “oxyntomodulin,” “glucagon-like receptor-1,” “peptide YY,” “insulin-like peptide-5,” “oats,” “*Avena sativa*,” and “weight lowering” has been extracted using following scientific web browsers “Google Scholar,” “Scopus,” “Science Direct.” First, in this article, obesity and the role of appetite hormones have been discussed in detail; afterward, the weight-lowering impact of oat consumption based on its various bioactive components has been discussed in detail.

## Obesity Causes and Risk Factors

**Fig. 1:**
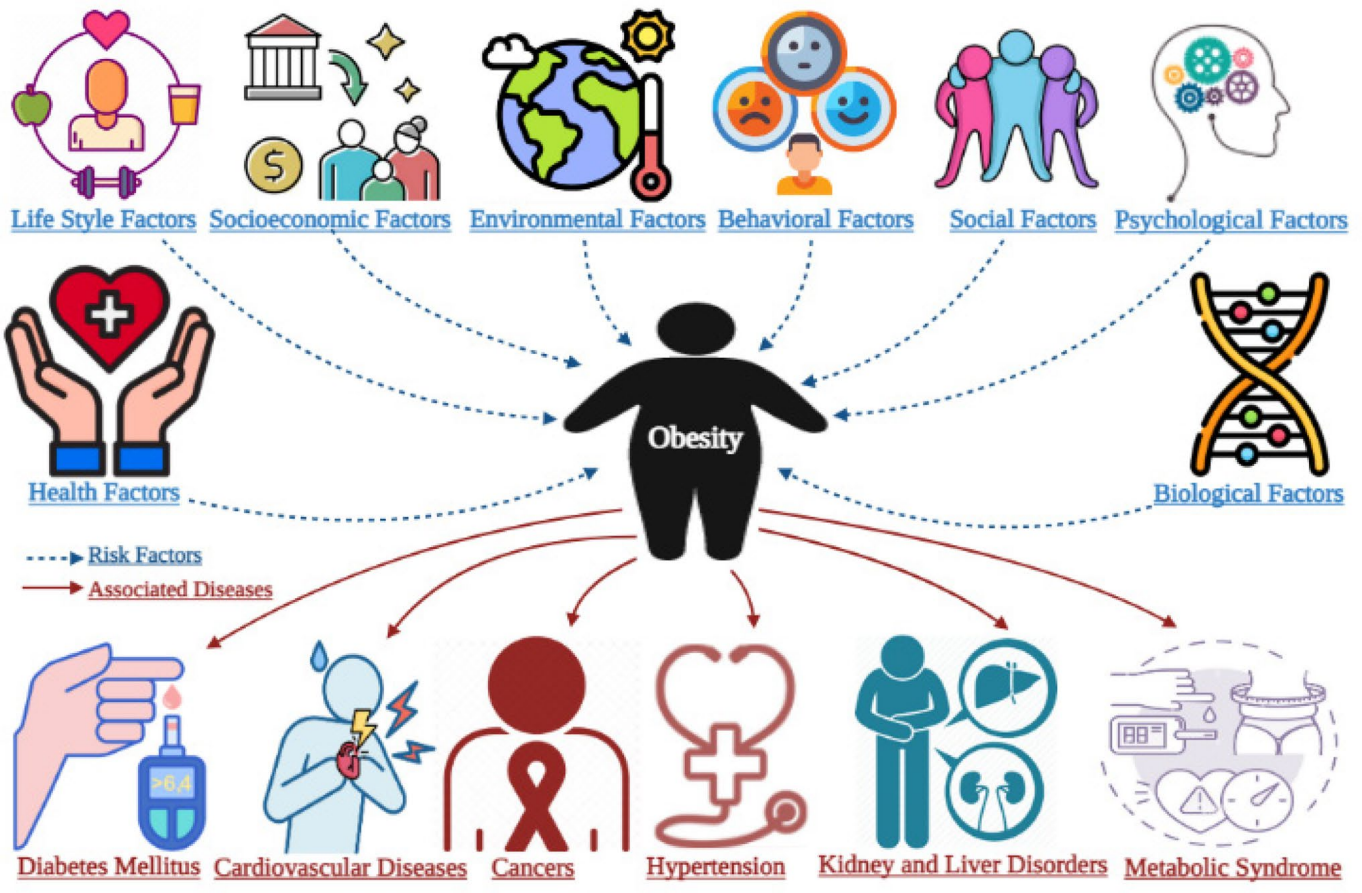
Risk factors and health complications associated with obesity (created with Biorender.com)

Obesity is a multifaceted illness with several causes [3] as elaborated in blue-dotted lines in Fig. 1. The most common risk factor for adiposity was fast-food consumption, with 61.67% of individuals reporting it, followed by eating three meals per day (58.89%), a sedentary lifestyle (53.33%), sleeping time (44.22%), hypercaloric nutrition (43.56%), excessive alcohol consumption (42.89%), and depression symptoms (31.78%). In 67.33% of cases, an unhealthy lifestyle was identified as a composite risk factor. Fast-food consumption increases the risk of obesity by 1.85 times, whereas sedentary living increases the risk by 1.79 times [7]. Recent research indicates that the environment has a significant role in the etiology of obesity and associated comorbidities. As a result, increased obesity, insulin resistance (IR), type 2 diabetes mellitus (T2DM), and lipid metabolism changes have been linked to air pollution, exposure to chemical substances that interfere with metabolism, excessive consumption of ultra-processed foods, changes in the intestinal microbiota, and sedentary lifestyle. These factors have a greater impact on some stages of life, such as the first thousand days, since they change the expression of genes that regulate hunger/satiety regulatory systems, energy expenditure, and adipogenesis [8]. Mutations in genes that play a major role in the central or peripheral control of energy balance identify rare hereditary obesity syndromes. These mutations cause the early onset of severe obesity and an insatiable appetite (hyperphagia), meaning that the genetic component may account for 40–70% of obesity. The functions of genes in the mechanisms that lead to obesity, on the other hand, are uncertain [9].

## Obesity-associated Health Complications

Being overweight and obese are two of the most frequent lifestyle disorders that create extra health issues and contribute to a variety of chronic diseases (as depicted with maroon lines in Fig. 1) such as T2DM, cancer, cardiovascular disease (CVDs), and metabolic syndrome (MetS) [2]. Obesity has a significant economic impact on the system of health care. Direct and indirect effects of obesity account for 10% of healthcare costs. Taking steps to prevent, manage, and treat obesity is expensive. Obese people spend 32% more on medical costs than people of normal weight. Obesity has a large number of short- and long-term complications, as well as a potential economic impact [10]. Obesity is on the rise globally, with roughly 20% of ICU patients suffering from it [1]. Adipose tissue is metabolically active, and visceral adipose tissue, in particular, has a negative adipocyte secretory profile, leading to IR, persistent low-grade inflammation, T2DM, hypertension (HTN), CVDs, dyslipidemia, obstructive sleep apnea, chronic kidney disease (CKD), nonalcoholic fatty liver disease (NAFLD) and hypoventilation syndrome, physical impairments of many kinds and mental problems [1]. Excess fat deposited in visceral adipose tissue and ectopic depots (such as muscle and liver) has also been related to increased cardiometabolic risk, as has a greater fat-to-lean mass proportion (e.g., normal-weight metabolically obese) [3].

## Obesity-associated Regulatory Hormones

**Fig. 2:**
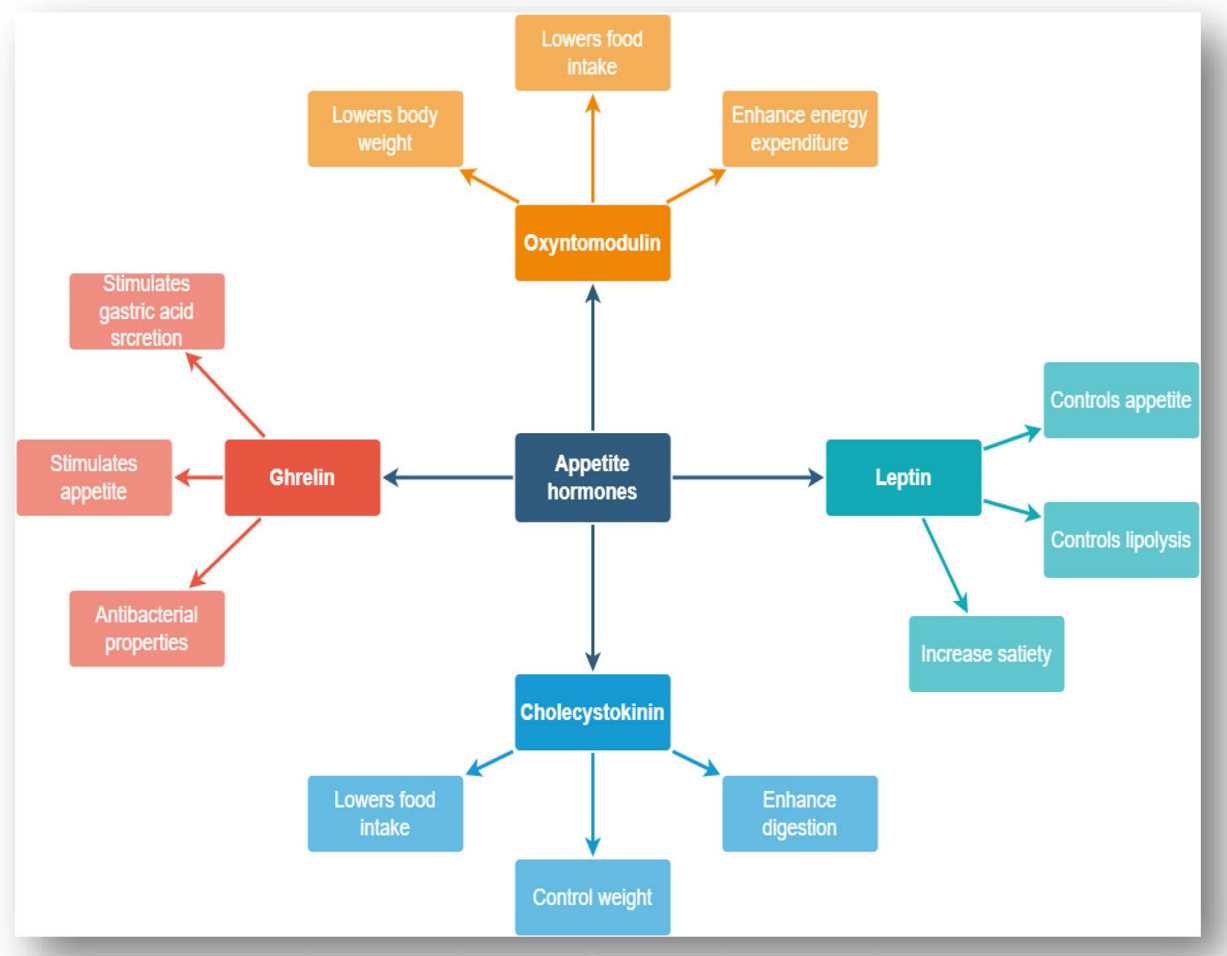
Role of various hormones on food regulation and weight management

Hormones that control hunger, satiety, obesity, glucose, and maintain weight include leptin, ghrelin, cholecystokinin (CCK), oxyntomodulin (OXM), glucagon-like receptor-1 (GLP-1), insulin-like peptide-5 (INSLP-5), and peptide YY (PYY). A brief description of these hormones in relation to their regulatory roles towards hunger, satiety, and body weight management (Fig. 2) has been discussed in this section.

### Role of Ghrelin in Obesity Control

Ghrelin is an endogenous ligand of the growth hormone secretagogue receptor (GHSR). It is a peptide hormone with 28 amino acids that have been acylated. Ghrelin has been isolated from the stomachs of both humans and rats, and it has also been found in the hypothalamus arcuate nucleus [11]. Ghrelin is also known as “the hunger hormone.” Ghrelin must be acylated, generally with octanoic acid, before it can bind to and activate the ghrelin receptor, which is a G protein-coupled receptor [12]. Ghrelin is responsible for appetite regulation, body weight management, learning, memory, cognition, reward, sleep, taste sensitivity, olfaction, and sniffing [12, 13]. It enhances cardiac function, decreases blood pressure, and protects the kidneys, heart, and brain by stimulating stomach acid production and motility. It enhances the utilization of carbohydrates as a fuel source while sparing fat, reduces lipid oxidation, and increases lipogenesis. It is analgesic, sympatholytic, antimicrobial, antifibrotic, and osteogenic. Ghrelin also enhances the secretion of prolactin, growth hormone, adrenocorticotropic hormone, glucagon, vasopressin, cortisol, and oxytocin; it also postpones puberty and lowers thyroid hormone and testosterone. Ghrelin protects the body in a number of ways, including suppressing damaging inflammation and activating autophagy [13].

Ghrelin receptors are present mostly in neurons that generate agouti-related protein and neuropeptide Y. According to previous studies, ghrelin and GHSR are implicated in the regulation of energy homeostasis and their administration can enhance food intake and body weight growth. Ghrelin activates AMP-activated protein kinase in the hypothalamus, resulting in reduced intracellular long-chain fatty acid levels. Ghrelin appears to impact food cue response via a neural network involved in eating control and appetitive response to food cues. It induces obesity by increasing the expression of fat storage-related proteins in adipocytes in addition to stimulating hypothalamic orexigenic neurons [11]. Scientific studies elaborating on the role of various appetite hormones in appetite regulation have been tabulated in Table 1.

**Table 1:** Mechanism of actions of various appetite hormones in appetite regulation

**Hormones**	**Mechanism of action**	**References**
**Ghrelin**	• Stimulates motility and gastric acid secretion • Enhances cardiovascular performance • Reduces blood pressure while also protecting the brain, heart, and kidney • Increases appetite • Encourages the use of carbohydrates as a fuel source while avoiding the use of fat • Lipogenesis is promoted by inhibiting lipid oxidation	[13]
**Leptin**	• Control the appetite, body mass, and reproductive function, as well as fetal development • Proinflammatory immune responses • Promote angiogenesis and lipolysis	[14]
**Cholecystokinin**	• Control insulin secretion • Have a therapeutic role in the treatment of type 2 diabetes and obesity	[15]
**Glucagon-like peptide 1**	• Cause satiety • Reduce appetite indirectly • Increasing insulin • Inhibiting glucagon secretion • Reduces stomach emptying and food intake • Restricting weight gain	[16] [17]
**Oxyntomodulin**	• Hypoglycemia effects and body weight reduction • Normalize adiposity, lipid metabolism, and hepatic steatosis	[18]
**Insulin-like peptide-5**	• Control appetite	[19]
**Peptide YY**	• Hunger suppression • Body weight management	[20]

### Role of Leptin in Obesity Control

Leptin is a peptide hormone that controls appetite, body mass, and reproductive function, as well as fetal development, lipolysis, angiogenesis, and proinflammatory immune response [14]. Leptin has long been known to regulate energy balance, neuroendocrine function, metabolism, and other physiological activities and it is also a pleiotropic protein. For satisfaction signals, it directly acts on the peripheral tissues and central nervous system (CNS) and is therefore known as “the satiety hormone.” The obese (ob) gene encodes leptin, which is released by adipose tissue. Leptin is a crucial mediator that controls both immunity and feeding. Leptin has the ability to influence both innate and adaptive immune responses. Dysregulation of cytokine production, increased vulnerability to viral infections, autoimmune disorders, malnutrition, and inflammatory reactions are all linked to leptin deficiency/resistance [15].

A protein that binds to leptin is known as a leptin receptor (LEP-R). Leptin’s pleiotropic activities are facilitated by LEP-R distribution, which controls body mass via a negative feedback loop between adipose tissue and the brain. Leptin resistance is characterized by decreased satiety, increased food consumption, and an increase in total body mass. Obesity is commonly the outcome, which reduces the efficiency of exogenous leptin as a therapeutic agent. Combining leptin treatment with leptin sensitizers may thus help overcome leptin resistance and, also obesity, as a result [14]. Leptin has been shown to successfully lower food intake and body weight and was first considered to be beneficial in the treatment of obesity. Obese persons, on the other hand, have high levels of circulating leptin and are insensitive to exogenous leptin therapy. Leptin resistance is defined as leptin’s inability to exert its anorexigenic effects in obese persons, and therefore leptin’s lack of therapeutic usefulness in obesity [16].

### Role of CCK in Obesity Control

 CCK is a hormone that mediates its biological functions by binding to and activating CCK-1 and CCK-2 receptors in the stomach, as well as neurons in the enteric and CNS. Yet, most of our understanding of CCK’s physiological relevance has centered on its ability to induce short-term satiety. CCK, on the other hand, has been found to play an important role in overall beta-cell function as well as insulin secretion and survival. As a result, enzymatically stable, physiologically active CCK peptide analogs with therapeutic potential in obesity and T2DM have been created. Moreover, several studies have related CCK’s metabolic and therapeutically relevant biological activities to those of the incretin hormones GIP (gastric inhibitory polypeptide) and GLP-1, as well as amylin and leptin. As a result, safe CCK compounds have the potential to be successful adjuvant therapy as well as standalone weight-loss and glucose-lowering medications [17].

Gibbs, Young, and Smith demonstrated in 1973 that exogenous cholecystokinin (CCK) lowers food intake in rats. CCK is produced by enteroendocrine I cells found throughout the gastrointestinal tract. When CCK binds to its receptor CCK1R, it activates vagal afferents, which send post-ingestive input to the hindbrain. The energy state influences the sensitivity of vagal afferent neurons (VAN) to CCK, and CCK signaling modulates gene expression of other feeding-related signals and receptors produced by VAN. CCK functions throughout the GI tract to enhance digestion and nutrition absorption, in addition to its satiating benefits [18].

### Role of GLP-1 in Obesity Control

GLP-1 has a number of metabolic effects, including glucose-dependent insulin secretion stimulation, decreased stomach emptying, food intake inhibition, increased diuresis, and natriuresis, as well as rodent b-cell proliferation regulation. GLP-1 also has cardioprotective, neuroprotective, anti-inflammatory, and anti-apoptotic properties, and it has implications for memory and learning, in addition, to rewarding palatability and behavior. GLP-1 receptor agonists have been effectively used in clinical trials for the treatment of T2DM, and various GLP-1-based pharmacotherapies are now being evaluated in clinical trials for the treatment of obesity [19].

As an adaptation to leptin resistance, intestinal L-cells released GLP-1 in response to increased postprandial energy levels. In obese people, GLP-1 caused satiety and reduced appetite indirectly [20]. GLP-1 is generated by enteroendocrine cells in the stomach that controls meal-related hyperglycemia by boosting insulin and decreasing glucagon release. GLP-1 also lowers stomach emptying and food intake, allowing for greater nutritional absorption while limiting weight gain [21].

### Role of OXM in Obesity Control

In humans, OXM inhibits appetite and decreases food consumption. In obese adults, OXM was found to be a dual agonist for the glucagon (GCG) receptor (GCGR) and the GLP-1 receptor (GLP-1R), suppressing hunger, increasing energy expenditure, and causing weight reduction [22]. Exogenous administration of OXM has been proven to lower body weight in people in several trials. The capacity of OXM to both lower food intake and enhance energy expenditure results in weight loss [23]. OXM is a product of the glucagon precursor proglucagon, which is synthesized and released by the stomach’s endocrine L-cells after enzymatic processing by the precursor prohormone convertase 1/3. It consists of the whole glucagon sequence plus a C-terminal octapeptide with a total of 37 amino acids and is equivalent to the proglucagon sequence 33–69. It possesses glucagon-like bioactivity, as one might assume, but it also stimulates the GLP-1 receptor, which is unusual. This has given the molecule a unique position as a GLP-1 co-agonist, which is generating a lot of buzzes right now because of its potential to treat diabetes and obesity [24]. To produce prolonged release in vivo, one particular analog with increased and balanced GCGR/GLP-1R activations was chemically linked with polyethylene glycol (PEG). In diet-induced obesity (DIO) animal models, the pharmacological effects of its PEGylated homolog were investigated. Chronic weekly dosing resulted in considerable hypoglycemia effects and body weight reduction, as well as normalizing adiposity, lipid metabolism, and hepatic steatosis, with dosage dependency. The analog has a lot of potentials to evolve into a new anti-diabetic and/or anti-obese contender based on its in vitro and in vivo characteristics [22].

### Role of INSL5 in Obesity Control

INSL5 is an orexigenic gut hormone found in a subset of colonic and rectal enteroendocrine L-cells, along with the anorexigenic hormones GLP-1 and PYY. Unlike GLP-1 and PYY, calorie restriction raises INSL5 levels [25]. INSL5 is hypothesized to function via the peptide receptor 4 of the RXFP4 (relaxin/insulin-like family). RXFP4, a G protein-coupled receptor (GPCR) with adenylyl cyclase inhibitory function, is located throughout the gastrointestinal tract. RXFP4 has also been demonstrated to raise Ca + 2 concentrations, indicating that it can form interactions with other G proteins [26]. RXFP4 has been connected to appetite control; in rats, combined RXFP4 and RXFP3 agonists evoked a rise in food intake following intra-cerebroventricular injection, which remained present at 4 and 24 at 4 and 24 h later [27].

### Role of PYY in Obesity Control

**Fig. 3:**
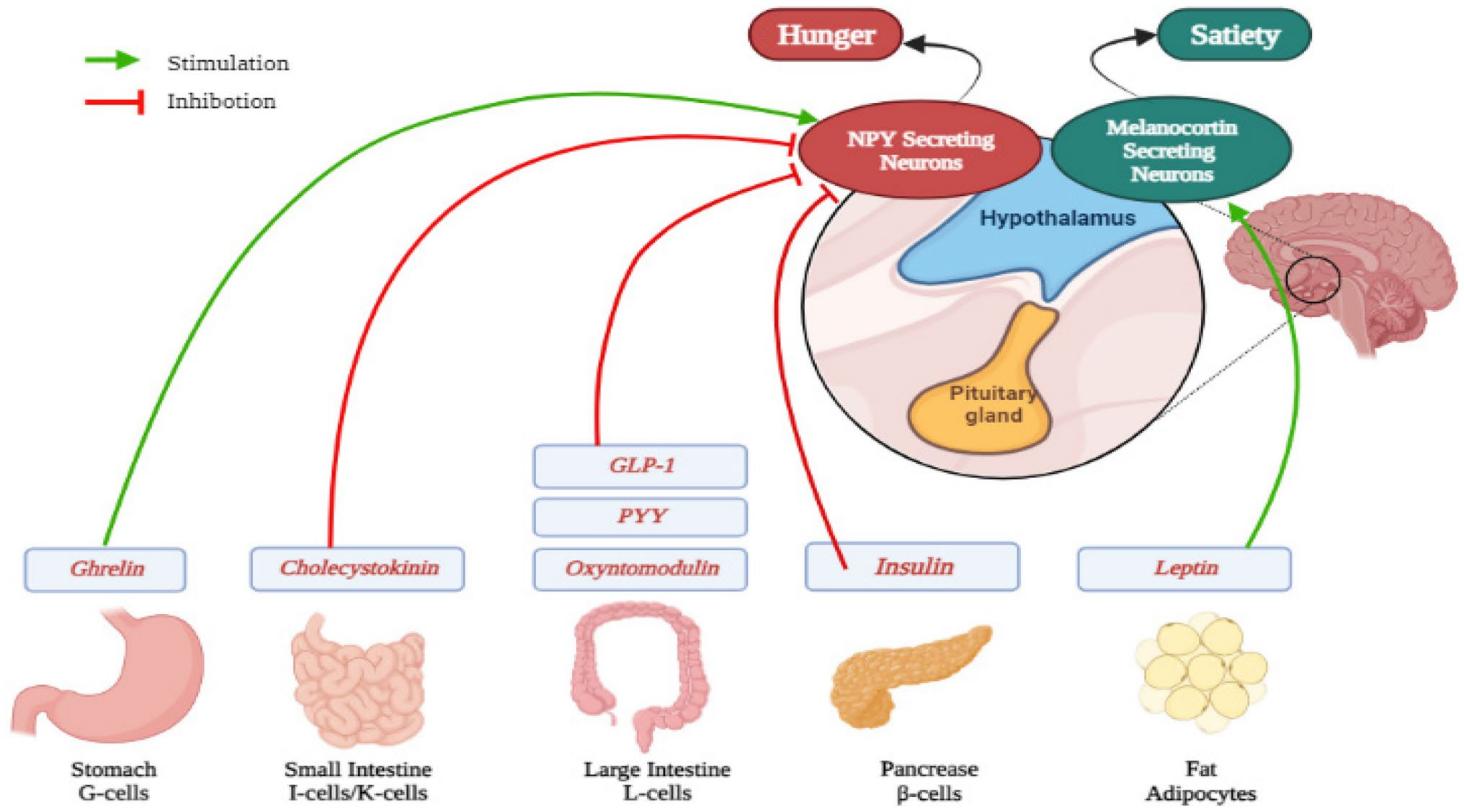
Various hormones associated with hunger and satiety (created on Biorender.com)

PYY activities were first studied for their local effects within the gastrointestinal system; it slows stomach emptying and inhibits gall bladder emptying [28]. So far, the majority of research on the influence of the gut hormone PYY on appetite suppression and body weight control has been conducted. PYY’s interaction with hypothalamic Y2 receptors is thought to be responsible for several physiologic effects [29]. PYY is secreted by GI tract L-cells and carried throughout the gut; immunoreactivity to PYY is low in the proximal small intestine but increases in the ileum and continues to rise in the large intestine towards the rectum [30]. The role of hormones in appetite regulation is depicted in Fig. 3.

## Oat (*Avena Sativa*) Consumption

Avena sativa, sometimes known as “oat,” is a Poaceae family plant. In the USA, the importance of this cereal ranks third following wheat and corn, and it ranks fourth globally. They are among the most extensively produced plants in terms of nutrients [31]. Oats cultivation has dramatically deteriorated over the twentieth century, as higher-yielding crops such as winter wheat and maize have replaced them [32]. Oat is a food crop and ancient grain that is grown and enjoyed all over the world. Its nutritional makeup and the multifunctional effects of certain bioactive components are gaining prominence [33]. Oats are a very nutritious and healthful grain that may be used in a variety of culinary items, based on their chemical qualities and mineral makeup [34]. Oat grains are an excellent source of nutrients, minerals, and phytochemicals that may be used as both nutraceuticals and food [35].

Oats have five certified health claims from the European Food Safety Authority (EFSA). Four of these address soluble fibers unique to oats, beta-glucans, and include blood cholesterol management, increased fecal volume, and better blood glucose balance. The sixth argument is that the high amount of unsaturated fatty acids, particularly in the endosperm, lowers the risk of heart and vascular disease. Oat starch also has a low glycemic index, which is beneficial for weight loss. Polyphenols and avenanthramides (AVNs) found in oats are antioxidants and anti-inflammatory. Oat products can be labeled gluten-free in the European Union (EU) (since 2009), the USA (since 2013), and Canada (since 2015) if the gluten contamination level is less than 20 ppm [32]. Beta-glucan is a dietary fiber component present in oat grains. It is the primary active ingredient in oats, having shown cholesterol-lowering and anti-diabetic properties. Oats also include phenolic acid, tocols, sterols, avenacosides, and AVNs, which are all healthy. Oats have been demonstrated to improve human health by strengthening immunomodulation and improving gut flora. Moreover, oat eating aids in the prevention of illnesses such as atherosclerosis, dermatitis, and some types of cancer [33]. It is also commercially nutritious. Beta-glucan, a soluble dietary fiber present in oat bran, oat grain, and oatmeal, helps lower blood cholesterol levels. Oat has been demonstrated to be a possible preventative agent for intestinal dysfunction, cancer, celiac disease, obesity, and other disorders. Because of the numerous health advantages they bring, their consumption has increased dramatically, and they have quickly become more popular [31].

### Nutritional Composition of Oats

Oats are one of the significantly nutritious cereals with a highly functional and nutraceutical profile, which have been consumed for centuries. Several evidential studies conducted on various oat varieties have proved their valuable composition (as tabulated in Table 2). Reported approximate oat moisture content was 4.21%, the content of ash was 1.97%, the nitrogen-free extract was 55.75%, crude fat was 6.91%, crude protein was 12.62%, and the total fiber was about 13.65% [34]. Another study reported percentage ranges for oat contents as moisture varying from 8.5 to 9.8, crude protein (11.9 to 15.8), crude fat (6.7 to 10.3), crude fiber (2.1–3.5), ash (1.2–1.3), and nitrogen-free extract (72.6–74.3%) [36]. Proximate composition of some oat varieties revealed protein content as 12.69% (Avon var.), crude fiber content as 17.83% (SGD-81 Var.), fat concentration as 6.67% (Avon Var.), moisture content as 9.29% (SGD-81 Var.), ash concentration as 6.02% (SGD-2011 Var.), and nitrogen-free extract as 60.78% (S-2000 Var.) [37]. Similarly, the reported contents for starch are 494 mg/g, total soluble sugars 5.3 mg/g, and the protein in total is 182.9 mg/g [38]. Hulled oats contain significantly larger amounts of insoluble and soluble dietary fibers and beta-glucans as compared to hulled wheat. This property makes it an important nutritional element [39].

**Table 2:** Proximate nutritional, mineral, and phytochemical profile of whole, naked, and sprouted oats

**Nutrient/component**	**Whole oats**	**Naked oats**	**Sprouted oats**	**References**
Moisture	11.95%	8.5–9.8%	-	[35, 36]
Ash	-	1.2–1.3%	-	[36]
Carbohydrates	53.35%; 55.75%	72.6–74.3%	-	[34–36]
Proteins	8.35–17.72%; 182.9 mg/g	11.9–15.8%	10.7%	[35, 38, 45]
Fats	7.88%; 6.91%	5.91–7.87%; 6.7–10.3%	-	[34, 36, 38, 42]
Oleic acid/oil	30.7–32.2%; 45–53%; 33.5–36.7%	-	-	[40–42]
Palmitic acid/oil	21.4–22.7%; 12–16%; 15.3–17.8%	-	-	
Linoleic acid/oil	34.6–38.2; 36–42%; 36.2–38.7%	-	-	
Fiber	13.65%;	2.1–3.5%	-	[34, 36]
Magnesium	2.89–7.62 mg/L; 115 mg/100 g; 1193.90–1352.88 mg/kg; 1166–1486 mg/kg	62.4–89.1 mg/100 g DM	-	[34–36, 44, 45]
Manganese	0.93–3.71 mg/L; 115 mg/100 g; 34.03–42.31 mg/kg; 30.0–49.3 mg/kg	-	-	[34, 35, 44, 45]
Sodium	3.71–8.03 mg/L; 177.08–249.97 mg/kg	-	-	[35, 44]
Copper	0.35–3.36 mg/L; 4.75–5.75 mg/kg	0.2–0.4 mg/100 g DM	-	[35, 36, 44]
Iron	2.15–6.82 mg/L; 9.23 mg/100 g; 45.58–63.84 mg/kg; 33.5–48.9 mg/kg	2.5–3.0 mg/100 g DM	-	[34–36, 44, 45]
Zinc	1.30–3.37 mg/L; 8.3 mg/100 g; 22.22–28.44 mg/kg	1.6–2.0 mg/100 g DM	-	[35, 36, 38, 44]
Potassium	50.70–59.60 mg/L; 337 mg/100 g; 3937.18–4645.44 mg/kg	241.7–258.3 mg/100 g DM	-	[34–36, 44]
Calcium	60.13 mg/100 g; 898.62–967.72 mg/kg	44.0–102.7 mg/100 g DM	-	[34, 36, 44]
Phosphorous	474.06 mg/100 g; 2342.40–3303.93 mg/kg	-	-	[34, 44]
Total soluble sugars	5.3%	-	-	[38]
Starch	494.3 mg/g	-	-	
Oxalates	-	28.2–71.4 mg/100 g DM	-	[36]
Phytates	-	269.6–293.0 mg/100 g DM	-	
Tannins	-	38.8–51.5 mg/100 g DM	-	
Beta glucan	-	-	2.1%	[43]
Thiamine	-	-	687.1 mcg/100 g	
Riboflavin	-	-	218.4 mcg/100 g	
Gamma amino-butyric acid	-	-	54.9 mcg/100 g	
Antioxidants	-	-	1744.3 mg TE/100 g	
Free phenolic	-	-	507.4 mg GA/100 g	

Fat contents of oat cultivars varied from 2.9 to 6.1%. The fat content of naked oat cultivars was substantially greater than that of hulled oat cultivars. Oleic acid (30.7–32.2%), palmitic acid (21.4–22.7%), and linoleic acid (34.6–38.2%) were the most frequent fatty acids in all oat cultivars studied [40]. Oleic acid (45–53%) was the most abundant fatty acid, followed by linoleic (36–42%) and palmitic (12–16%) [41]. The fatty acid concentration of acyl lipids from seven naked oat cultivars revealed that raw lipid content in the grain ranged from 5.9 to 7.87%. Linoleic (36.2–38.7%), oleic (33.5–36.7%), and palmitic (33.5–36.7%) are the primary fatty acids found in the naked oat lines studied (15.3–7.8%). Because of the content of oleic and linoleic fatty acids and their ratio, the lipids of naked oats fall into the oleic-linoleic group of vegetable oils (1:1) [42].

Among these minerals, Zn [37], Mn, Mg, and Fe, Cr, and Zn were in significant amounts [43]. In oats, the Zn level is greater than the Fe content [38]. Another study reported minerals Fe, Cu, Zn, Mg, Ca, and K in oats [36]. A similar study conducted on a comparison of various oat varieties revealed the presence of K, P, Mg, Ca, Na, Fe, Mn, Zn, and Cu [44]. Likewise, the results of another study indicated the Ca, Ph, Mg, Fe, and K [34]. Regarding tannin, saponin, and phytic acid, eleven genotypes had minimal antinutritional components [38]. The concentrations of tannin, phytate, and oxalate varied from 38.8 to 51.5, 269.6 to 293.0, and 28.2 to 71.4 mg/100 g respectively [36]. Protein, essential amino acids (Phe, Met, Cys), minerals (Mg, Zn, Fe, Ca), riboflavin, and polyunsaturated fatty acids (especially cis-11-eicosanoid, palmitoleic, alpha-linolenic, linoleic acids) have all been found in sprouted oat powder. As a result, sprouted oat may be used as a gluten-free component with increased nutritional and bioactive qualities [45]

### Bioactive Component of Oats

Oats are high in phenolic acids and AVNs, both of which are helpful to health [46]. Oats have strong antioxidant potential and great fiber, beta-glucan, lysine, thiamine, 4-amino butanoic acid (GABA), and free phenolic compound contents, making them a useful nutritive and functional component. In sprouted oat powder, increased protease and alpha-amylase activities were discovered, as well as decreased lipase activities, which are promising traits for improving its nutritional, sensory, and health-promoting aspects [45]. Oat has the largest concentration of free phenolics (up to 30% of total phenolics) with ferulic acid as the main phenolic acid in it. These contents can be reduced during the removal of the husk [39].

Flavonoids present in oats ranged from 754.16 to 1056.66 mg of quercetin equivalent (QE), total phenolic content (TPC) of oats ranged from 36.07 to 59.6 mg of gallic acid equivalent (GAE), percent scavenging activity 24.33–55.88%, and anthocyanin concentration ranged from 0.5 to 2.87 mg of cyanidin-3-glucoside (C3G)/kg and total flavonoid content (TFCs) 663.75–697.5 mg QE [37]. The soluble phenolic fraction of the oats revealed phenolic aldehydes, phenolic acids, AVNs, mono-, and diglycerides [47]. In decreasing order of abundance, seven phenolic acids and one phenolic aldehyde were found in oats, including cinnamic, ferulic, p-coumaric, vanillic, syringic, 2,4-dihydroxybenzoic, syringaldehyde, and o-coumaric acids. The cumulative concentration of phenolic acids ranges from 1202 ± 52.9 to 1687 ± 80.2 mg/kg, and that of AVNs varies from 26.7 ± 1.44 to 185 ± 12.5 mg/kg. The vast bulk of phenolic acids was found as bound compounds [48••]. There were phenolic aldehydes, phenolic acids, and a ferulic acid dehydrodimer identified in the bound phenolic fraction [47].

Table 3 elaborates on the recent findings on bioactive components identified in oats. In 22 commercial oat products, including flaked oats, oat bran, oatcakes, rolled oats, and oat bran concentrate, eleven bound and thirteen free + conjugated phenolic acids and AVNs were discovered. In total, 16.7 mg TPCs (15.17 mg bound, 1.53 mg free + conjugated) and 1.2 mg AVNs were found in 11 g of oat concentrate. The different products had comparable compositions and quantities of the components, with ferulic acid (58–78.1%) being the most common [46]. Ferulic acid (53.6 μg/g oil), vanillin (43.33 μg/g oil), vanillic acid (0.78 μg/g), and coumaric acid (2.2 μg/g oil) were shown to be polyphenols [41].

**Table 3:** Bioactive components of oats

**Main group**	**Bioactive components**	**References**
**Oat**	Quercetin, ellagic acid, rutin, malic acid, pyrogallol, mandelic acid, catechin hydrate, morin, epigallocatechin gallate	[35]
**Sprouted oat flour beverage**	Free phenolic compound and high antioxidant activity	[49••]
**Oat**	Gallic acid, quercitin, flavonols, and anthocyanins	[37]
**Oat**	Free and unbound phenolics and ferulic acid is dominant	[39]
**Oats**	Soluble and insoluble phenolic components, ferulic acid, sinapic acid	[50]
**Commercial oat products**	Bound phenolic acid, free and conjugated phenolic acid, avenanthramides, ferulic acid	[46]
**Oat oil**	Vanillic acid, vanillin, ferulic acid, coumaric acid gallic acid, and polyphenols	[41]
**Oats**	Content of gallic acid, chlorogenic and ferulic acid increases, insoluble phenolic content	[51]
**Colored oats**	Phenolic aldehyde, avenanthramides, mono and diglyceride derivative of ferulic, caffeic, phenolic acid, p-coumaric acid, ferulic acid dehydrodimer	[47]
**Husked oat**	Ferulic, p-coumaric, cinnamic, vanillic, syringic 2,4-dihydroxybenzoic, syringaldehyde and o-coumaric acids, avenanthramides	[48]

Oat extracts demonstrated a concentration-dependent antioxidant response when tested as 2,2 diphenyl-1-picrylhydrazyl (DPPH) and 2,2-azino-bis-3-etylbenzothiazoline-6-sulfonic acid (ABTS) and free radical inhibitors. [35]. DPPH radical scavenging ability of oat revealed that the hulled oats had higher antioxidant activity than the ground oats, as phenolic content is elevated in the hull [47].

## Role of Oat/Beta-glucan on Appetite Hormone and Obesity

Many clinical investigations have been conducted to define the fate of oats in obesity prevention, lowering body weight, regulating appetite hormones, and enhancing insulin sensitivity. The results of such studies are compiled in Table 3 and are discussed sequentially hereafter. Many of these studies found interrelationships among oat or beta-glucan consumption with obesity, adiposity, appetite hormones, anthropometrics, percent body fat, BMI, lipid profile, satiety perception, stomach emptying, gut hormones, gut microbiota, and short-chain fatty acids [49••].

**Fig. 4:**
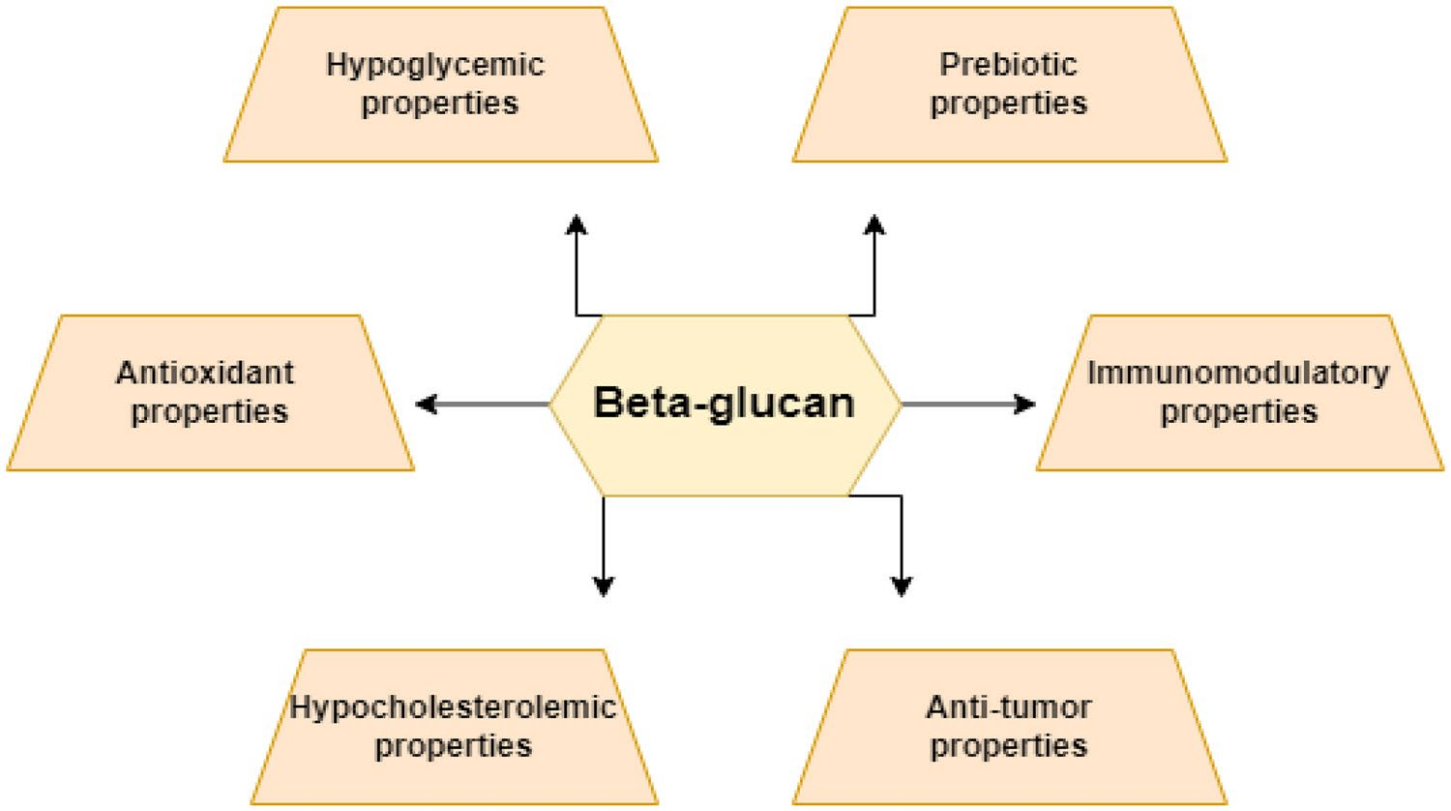
Bioactive potentials reported from oat beta-glucan

Beta-glucans are natural compounds with no substantial adverse effects. These come in two forms, insoluble and soluble, and they can interact with lipids and bile salts in the intestine, lowering cholesterol levels. These might be developed as a viable alternative therapy [50]. Figure 4 briefly explains the bioactive potentials of beta-glucan. Because of its multifunctional and bioactive properties, beta-glucans and soluble fibers are grown. The health advantages of beta-glucans are based on their capacity to ferment and create high-viscosity solutions in the human colon. Beta-glucans are significant molecules for lowering postprandial glucose and insulin responses, and several mechanisms have been proposed to explain their effects. Glycaemic regulation is influenced by dosage, length of intake, physicochemical properties, processing techniques, and dietary shape. Long-term use of beta-glucan at 3 g/day has been found to give additional advantages in diabetes treatment [51]. The relationship between oat beta-glucan and glycemic management, appetite-regulating hormones, and microbiota in T2DM was investigated. In 37 T2DM patients, the supplementation of a regular diet with oat beta-glucan (5 g/day) for 12 weeks resulted in significantly decreasing HbA1c, insulin, C-peptide, homeostatic model assessment (HOMA), *Lactobacillus* spp. and butyrate-producing bacteria. The levels of leptin, GLP-1, and PYY were also different and proved the enhanced satiety levels [52••]. A recent study also looked at how two types of oat beta glucans and decaffeinated green coffee bean extract (GCBE) altered biomarkers related to obesity in overweight/obese individuals. For 6 weeks, four groups of participants were given a nutraceutical drink comprising 3 g or 5 g doses of 35% or 70% beta-glucan coupled with a fixed quantity of GCBE giving 600 mg/d1 of phenols twice a day. Food consumption, anthropometry, and other cardiometabolic indicators were measured. The intervention resulted in positive changes in TC, LDL-C, VLDL-C, TAGs, aspartate aminotransferase (AST), alanine aminotransferase (ALT), insulin, hemoglobin A1c (HbA1c), total body fat percentage (TBF), waist and hip circumferences, visceral fat percentage, and systolic blood pressure (SBP). Results indicated that a 5 g dose of 70% oat beta-glucan therapy reduced the greatest TBF percent and was proved helpful in assisting weight loss [53••].

Similarly, another study investigated the effect of oat on hunger, glycemia, and insulinemia in 33 adults of normal weight (22 female/11 male, mean age (year): 26.9 ± 1.0, BMI: 23.5 ± 0.4) being studied. Ad libitum, they were administered 4 g of MW (high molecular weight) oat beta-glucan. Subjective hunger, glycemia, insulinemia, and plasma GLP-1 responses were studied. At regular intervals, blood samples and subjective hunger evaluations were taken postprandially. The oat beta-glucan supplement increased feelings of satiety and fullness but had no effect on energy or food consumption during the ad libitum test meal. Blood glucose, plasma insulin, and plasma GLP-1 all had a treatment by time interaction. When compared to the control meal, GLP-1 was significantly lowered after 90 min, blood glucose at 30 min, and plasma insulin at 30 and 60 min, respectively). These results indicated that the 4 g of oat beta-glucan of high molecular weight MW helped suppress hunger and improves postprandial glycemia; however, it has no effect on plasma GLP-1 secretion [54••]. A 71-day clinical investigation on 14 dogs fed with 1% beta-glucan resulted in higher fecal output, lower fecal consistency, lower interleukin-4 serum concentrations, lower serum concentration of total cholesterol (TC), low-density lipoprotein cholesterol (LDL-C), and very low-density lipoprotein cholesterol (VLDL-C) [55•].

In HFD-caused hyperlipidemic rats, oat beta-glucan lowered body weight gain inhibits hepatic adipocyte hyperplasia, and decrease epididymal fat pad. In both liver and fat tissues, these effects were linked to the downregulation of FAS and SREBP-1, the upregulation of PPAR, and the stimulation of AMPK (AMP-activated protein kinase signaling). Oat beta-glucan partially reduced lipogenesis, activated AMPK reduced the production of lipid metabolism-related proteins such as sterol regulatory element binding protein (SREBP-1), Fas cell surface death receptor (FAS), peroxisome proliferator-activated receptors (PPAR), carnitine palmitoyltransferase 1 (CPT-1), and activating acetyl-CoA carboxylase (ACC), which are AMPK’s downstream targets. These findings demonstrate that oat beta-glucan administration lowers lipid levels in HFD mice via the AMP-activated protein kinase (AMPK) signal pathway, suggesting a potential use for the prevention and treatment of CVD and obesity [56•].

The early effects of oat supplementation in 106 obese women (mean BMI 37.73 kg/m^2^) on a hypocaloric diet for 8 weeks for the treatment of metabolic issues associated with obesity. As a result, the mean anthropometric measurements of waist-to-hip ratio (WHR), waist circumference, body fat percent, and systolic blood pressure (SBP) were drastically reduced. This study supported the beneficial effects of a dietary oat supplement on central obesity, percentage of body fat, and several metabolic disorders [57]. Another controlled clinical trial was conducted on 62 hypercholesterolemic men and women 18–65 years of age by feeding 80 g/day oatmeal revealed a consistent relationship between micro-organism modification, the significant amelioration of hypercholesterolemia by lowering TC, LDL-C, and apolipoprotein B [58].

Oats and oat derivatives have been consumed for generations and are a common nutritional staple. They are high in nutrients, vitamins, minerals, and other physiologically active compounds, giving them a wide range of health benefits. These goods are widely used in numerous industries due to their great nutritional value. This article’s goal is to present the ongoing state of knowledge on the impact of oat products on human health as well as their industrial uses. The link between consuming oat-based goods and the development and management of conditions such as T2DM, CVDs, and obesity is highlighted in particular. Many research findings show that including oat products in one’s diet has beneficial benefits in the context of the aforementioned illnesses, owing to the high quantity of water-soluble dietary fiber, particularly beta-glucans, which demand special attention. According to scholarly research, greater consumption of the latter is connected to improved glycemic management, and lower blood cholesterol, and may also help people lose weight who are overweight or obese. Oat products, for these reasons, demand special attention and should be advised for both preventative and therapeutic use in metabolic diseases [59]. C57-Bl mice were randomly assigned to a chow diet (N) group, a HFD group, and one of three dosages of oat beta-glucan (high beta-glucan, medium beta-glucan, and low beta-glucan). In obese mice, oat beta-glucan increased intestinal peptide Y-Y and plasma peptide Y-Y expression [60]. The consumption of liposomes produced by fractionated oat oil is thought to alter digestion and postprandial lipemia, as well as promote satiety. Before and four times after the meal, blood samples were examined for insulin, glucose, plasma lipids, and intestinal hormones (GLP-1, GLP-2, PYY, CCK) in 19 subjects who consume 35 g of lipids from yogurt and liposomes produced by fractionated oat oil in breakfast. According to this study, liposomes produced by fractionated oat oil consumption may delay fat digestion, modify postprandial plasma lipids, and influence satiety. The impact of liposomes produced by fractionated oat oil on GLP-2 suggests that liposomes produced by fractionated oat oil use can also benefit intestinal health [61]. Consumption of oat-glucan-supplemented diets to create targeted short-chain fatty acids (SCFAs) in vivo might be a possible method for lowering fat mass buildup and a tool for managing obesity [62]. After the standardized meal, the polar lipid resulted in higher quantities of the gut hormones GLP-1 and PYY compared to rapeseed oil (RSO). The findings show that oat polar lipids may have nutraceutical effects by influencing acute and postprandial metabolic responses [63]. The gastrointestinal systems that mediate satiety are influenced by the viscosity produced by oat beta-glucan. Old-fashioned oatmeal and instant oatmeal (IO) compared to ready-to-eat breakfast cereal (RTEC) increased appetite control over 4 h The initial viscosity of oats may be especially essential for appetite reduction [64]. The bread was enriched with beta-glucan or resistant starch (RS) to investigate possible postprandial advantages in terms of gastrointestinal hormone responses. There were no significant changes between the two breads in ghrelin, GLP-1, PYY, or insulin response. A much-decreased urge to eat and a higher level of fullness were observed 15 min after beta-glucan and RSB ingestion and up to 180 min later compared to the reference food [65]. Seven male and seven female volunteers of BMI 25–36 kg/m ate five breakfasts (various dosages of b-glucan), and their nutritional intake was evaluated 4 h later. Blood was drawn to evaluate ghrelin, cholecystokinin levels, glucose, insulin, and visual analog scales were used to assess subjective satiety. The mechanism through which beta glucan promotes satiety is believed to include the release of cholecystokinin [66]. We expected that increasing dosages of beta-glucan would raise plasma PYY levels in overweight human adults. An increase in the dose of beta-glucan resulted in greater plasma PYY levels from 2 to 4 h after the test meal, with significant variations between groups [67]. Table 4 elaborates on the clinical studies done to investigate the impact of oats/beta-glucan consumption on appetite hormones and weight management.

**Table 4:** Impact of consuming oats/beta-glucan on appetite hormones and weight management

**Product/dosage**	**Subjects/study**	**Methodology**	**Results**	**References**
Green coffee phenolic extract and oat beta-glucans	• 60 obese humans • Dose–response randomized parallel trial	• 72 h record of food intake • Blood samples • Lipid profile determination • Blood pressure measurement • Anthropometry measurement	• Prevent obesity • Prevent type 2 diabetes • Reduce the cardiovascular risk • Lower total body fat percentage	[56•]
Green coffee phenols and beta-glucan	• 29 humans • Randomized cross-over blind trial	• Measurement of body weight and BMI • Percentage body fat • Skinfold measurement • Intracellular and extracellular water • Body circumferences measurement • 72 h dietary record	• Not reduce weight without modifying dietary and physical exercise habits • No change in body composition	[68]
Oat beta-glucan supplement	• 37 obese humans • Randomized double-blind clinical trial	• Fasting glucose • Insulin • HOMA • C-peptide • HbA1c • ghrelin • Lipid profile leptin • GLP-1 • PYY • Caloric intake • Intestinal microbiota	• Increase the feeling of satiety • Improve glycemic control • Modify gut profile	[55•]
Low molecular weight barley beta-glucan + High molecular weight beta-glucan	• 24 mice	• Serum biomarkers • Gut microbiota assessment • The concentration of short chain fatty acid • Measure absorption of fat • Real-time PCR	• Decrease in serum leptin • Decrease in LDL concentration • Reduction in messenger RNA expression of sterol regulatory element binding protein 1-c	[69]
Oat polar lipids	• 20 healthy persons	• Volunteers drank four liquid-based cereal test drinks • Test variables were assessed while fasting, 3 h after breakfast, and 2 h after a standardized meal	• Higher quantities of the gut hormones GLP-1 and PYY	[66]
Beta-glucan enrichment in bread	• 10 healthy adults	• Blood samples collected • Ghrelin • GLP-1 • PYY • Insulin • Glucose • Glycemic index	• Higher level of fullness • No change in GLP-1, PYY and ghrelin	[70]
Combining beta-glucan and whey protein in energy drinks	• 10 healthy females • Single blinded cross-over study	• Adaptive visual analog scale • Blood samples • Glycemic index • Acceptability • Palatability • BMI • Stadiometer	• Palatability and acceptability were similar • Drinks reduce postprandial blood glucose • Seen no effect on satiety and glycemic index • Natural sources are a good option	[71]
Beta-glucan	• 28 humans • Randomized placebo-controlled crossover trial	• Subjective appetite • Glucose • Insulin • Ghrelin • Gastric emptying • Peptide tyrosine assessed	• Effect on postprandial glucose • Effect on insulin • Effect on gastric emptying • No significant effect on appetite and food intake irrespective of the viscosity	[72]
Oatmeal	• 62 Hypercholesterolemic human • Randomized clinical trial	• Blood samples • Fecal samples • Lipid profile • Microbiota ribosomal RNA amplicon sequencing	• Lower cholesterol • Positive effect to firmicute phylum	[61]
Viscous dietary fiber (beta-glucan)	• 3877 humans • Randomized control trial	• Viscous fiber • Ad libitum diet • Body weight • Calorie restriction • BMI • Waist circumference, body fat	• Improve body weight • Reduce BMI • Reduce waist circumference	[73]
Oatmeal	• 5876 children	• National Health and Nutrition Examination Survey • 2015 USDA Healthy Eating Index • Dietary Research Food and Nutrient Database • Database of food pattern equivalents	• Children consuming oatmeal have good dietary quality and increase intake of essential nutrients	[74]
Oatmeal-based diabetes-specific nutritional formulas	• 22 humans with type 2 diabetes	• Amylin • Cholecystokinin • Ghrelin • Glucagon • Leptin • Peptide-YY	• Increased peptide YY and glucagon secretion	[75]
Oat	• 8 rats	• Compare and contrast the effects of oats (cereals) and soybeans (legumes), which are high in distinct types of NSP, on hunger regulation and fat storage in rats	• Short-chain fatty acids (SCFAs) in vivo lower fat mass buildup and manage obesity	[65]
Oat beta-glucan	• 14 dogs	• Plasma concentration of peptide YY ghrelin • Serum concentrations of glucose • Total cholesterol lipoprotein • Total tract apparent macronutrient digestibility • Immunoglobulin variables measured	• Reduce total blood concentration of cholesterol • Reduce the number of red blood cells • Lower concentration of interleukin-4	[58]
Oat beta-glucan	• 40 participants • Randomized crossover design	• Fasting blood sample • Apid visco-analyzer • Automatic glucose analyzer • Blood samples	• 0.4 g addition of oat beta-glucan in instant meal decreases glucose peak rise by 20%	[76]
Oat beta-glucan	• 33 normal-weight humans • Randomized double-blind cross-over design	• Blood samples • Blood glucose determination • Bohin rheometer • Plasma insulin • Plasma GLP-1	• Reduces appetite • No reduction is seen in ad libitum eating • Regulates postprandial glycemia • No rise in secretion of plasma GLP-1	[57]
Oat flour	• 106 obese women	• 24-h dietary recall • Group A consumed snacks while group B consumed a low caloric balanced diet • Anthropometric measurement	• Reduce central obesity • Reduction in the percentage of body fat • Reduction in metabolic disorders related to obesity	[60]
Oat beta-glucan	• 40 mice	• Hyperlipidemic mouse model • Oleic acid based hepG2 cells model	• Lipid-lowering effect through AMPK signal pathway	[59]
Oatmeal breakfast	• 50 humans • Randomized crossover clinical intervention	• Fasting blood samples • Visual analog scale • Weight • Height • BMI • High-density lipoprotein • Low-density lipoprotein • Waist circumference • Triglyceride • Nutrition data system • Plasma ghrelin concentration	• Increase satiety • Increase in cholesterol • No change in glycemic index • Glycemic load decreases • No change in HDL\LDL ratio • No change in triglyceride • No change in liver enzyme • Increase in HDL • Increase in LDL	[77]
Liposomes fractionated oat oil	• 19 healthy persons	• Blood sample analyses • GLP-1 • GLP-2 • CCK • PYY	• Influence satiety • Delay fat digestion • Modify postprandial plasma lipids • Improve the health of gut	[64]
Oat beta-glucan	• 48 subjects	• At least a week apart, subjects ingested isocaloric morning meals with instant oatmeal, old-fashioned oats, or RTEC in random sequence	• The initial viscosity of oats may be essential for appetite reduction	[67]
Oat cereal beta glucan	• 60 mice	• HDL cholesterol • Plasma neural peptide Y • Arcuate neural peptide Y • mRNA • Total cholesterol	• Rise in intestine peptide YY and Plasma peptide YY	[63]
Oat beta-glucan	• 14 subjects	• Solubility, viscosity, and molecular weight of beta-glucan were measured • Visual analog scale calculated the satiety	Promotes satiety • Cholecystokinin release	[78]
Oat beta-glucan	• 14 humans	• Volunteers were given a control meal and three cereals with varied beta glucan concentrations and blood samples were taken over 4 h	• An increase in the dose of beta-glucan resulted in greater plasma PYY levels from 2 to 4 h after the test meal	[79]

## Conclusion

Oats are rich in macronutrients, soluble fiber, minerals, vitamins, and several phytochemicals that have a positive role in maintaining body weight and BMI, reducing percent body fat, and regulating appetite and energy. The minerals, fats, and bioactive components that are commonly present in oats are Fe, Zn, Mg, Mn, oleic acid, palmitic acid, linoleic acid, ferulic acid, free phenolics, polyphenols, beta-glucan, and AVNs. Beta-glucan being the most active component plays a key role in weight management. Oat consumption in any of its desired forms, or especially the supplementation of beta-glucan, could enhance the satiety level by posing a considerable difference in is also seen in leptin, GLP-1, and PYY levels. The whole scenario lowers the appetite, postprandial glucose, and body weight. It also manages the gut microbiota. Conclusively, many studies support the scientific evidence that oats are beneficial in maintaining weight and have a positive impact on appetite hormones. But further scientific studies are required to support the potential of selective bioactive components of oats in appetite management especially in controlling satiety signals.
